# Accelerating health disparities research with artificial intelligence

**DOI:** 10.3389/fdgth.2024.1330160

**Published:** 2024-01-23

**Authors:** B. Lee Green, Anastasia Murphy, Edmondo Robinson

**Affiliations:** ^1^ Department of Health Outcomes and Behavior, Moffitt Cancer Center, Tampa, FL, United States; ^2^ Center for Digital Health, Moffitt Cancer Center, Tampa, FL, United States

**Keywords:** health disparities, artificial intelligence, machine learning, equity, bias

## Introduction

Health disparities are one of the most pressing health issues we face today, transcending the boundaries of any single profession or discipline despite many decades of research and novel interventions (1) This issue has shown stark divisions in equitable access to high-quality healthcare based on race, ethnicity, gender, language preference, disabilities, socioeconomic status, environment, and other factors reflecting systemic biases and structural health inequalities. Addressing these social determinants of health is crucial to reducing health disparities and achieving health equity.

Health disparities are a complex issue, as there are multiple contributing factors that extend well beyond individuals’ behaviors and choices (2). This multidimensional issue is based on historical, societal, and economic conditions. As a result, disparate health outcomes are seen among various population groups, including minorities or marginalized people or communities, and those without access to resources. The US healthcare system can perpetuate inequality by allowing differential access to quality healthcare, diagnostic delays, treatment inaccessibility, and unequal treatment between populations.

Technological tools arising from artificial intelligence (AI) have the potential to unravel the difficulties of addressing social determinants of health and mitigating health disparities (3). Mitigating health disparities is not just a mere assertion of AI's potential. Still, it is based on the robust body of literature affirming that AI is a transformative force that can be used to dissect the multifactorial social, genetic, and environmental factors of health disparities (3). We argue that using AI to address health disparities is not just a simple choice but a scientific imperative (4–8). As this discourse continues, we must recognize the pressing need for AI as an important aspect in our collective endeavor to alleviate health disparities. As such, we present a call to action to position AI as a critical tool in the pursuit of health equity.

## AI as a tool to address health disparities

AI is a rapidly developing field of study with incredible potential to change how we deliver care and provide health services (9). AI is already being used to address some of the most pressing health challenges and healthcare obstacles that we face as a society, from improving diagnostic accuracy to supporting precision medicine (10). Given the context of AI's preexisting utility in healthcare, AI technologies have revolutionary potential as a disruptive partner in addressing health disparities.

By taking advantage of these powerful tools, we can become more efficient and comprehensive, creating a paradigm shift revolutionizing how we measure, understand, and develop interventions to address disparities. Traditional approaches to address health disparities often fail to capture the issue's complexity and leave many gaps unresolved (11). By analyzing large datasets for patterns, correlations, or predictive markers that are difficult to identify through traditional analyses, AI can help us uncover hidden mechanisms and underpinnings of health disparities (12, 13). AI has many advantages compared with traditional strategies for addressing health disparities, notably in its ability to uncover unexpected correlations and relationships that have remained unidentified in human-driven analyses, offering new insights. By examining variables like genetic markers, environmental exposures, social determinants, and zip codes, AI can uncover novel connections that challenge current paradigms, revealing new areas for research and interventions (14).

## Challenges to AI

Though AI holds great promise as a tool to combat health disparities, it also presents some unique challenges that need to be considered.

First, AI is limited to the quality of data it’s trained on (5, 15, 16). Populations that have been historically marginalized and experience barriers to care are underrepresented in historical datasets, leading to them being negatively impacted when biased data and algorithms are deployed in real-world applications (15, 17, 18). If AI is implemented without regard for these biases, it can reinforce or even worsen existing disparities. There is a need for more inclusive datasets that accurately reflect the health experiences of various marginalized social, racial, and ethnic groups.

Second, equity must be considered during all stages of AI use and processes (7); it is critical to design algorithms with equity in mind. Technical approaches to mitigating discrimination and bias in algorithm development, like the open-source AI Fairness Project, should be used when applicable (https://aif360.res.ibm.com/). When health disparities are not considered during all stages of AI development and implementation, AI can perpetuate existing biases and inequalities within healthcare, which can disproportionately impact marginalized populations.

Third, there is a lack of diversity and representation within the teams developing and deploying AI algorithms (19). Greater representation from various stakeholders from diverse backgrounds, including policymakers, researchers, clinicians, and ethicists, must be included to ensure different voices and perspectives are included in the research team (20–22). Diverse research teams are more able to identify potential disparities, inequities, or underrepresentation that could lead to biased (15) AI algorithms (22).

Fourth, ethical standards and guidelines must be established around the use of AI. As previously mentioned, some critics contend that AI's dependence on historical data may perpetuate biases present in past healthcare practices (23); as such, it will be important to address this concern so that we avoid reproducing patterns that could be seen as discriminatory. Even if the models are built and algorithms developed a way that mitigates bias, there should still be a focus on ensuring that the algorithm output's use does not exacerbate disparities. Ethical standards should focus on mitigating bias, increasing transparency, and ensuring accountability regarding the creation and usage of AI algorithms.

Fifth and finally, there are concerns that becoming overly reliant on AI may undermine the clinician-patient relationship (14). Though algorithms cannot and should not replace human interactions in healthcare, incorporating AI as a decision support tool rather than a replacement for humans can harness and balance both the physician's expertise and machine's insights. Also, AI models or algorithms may potentially help enhance the patient-provider relationship by identifying key subject areas or talking points based on individualized patient factors, leading to better conversations and outcomes (24). AI should be designed to make the jobs of humans in healthcare better and easier. By recognizing concerns around AI, we can employ rigorous strategies, such as debiasing the data, optimizing development of algorithms, and providing ethical oversight, to ensure the integrity of both the data and the output before deploying AI strategies.

## Recommendations for leveraging AI to address health disparities

AI will present new challenges when it is used to address health disparities, but its potential is immense. Combining human ingenuity with AI offers a new roadmap towards an equitable healthcare system and overall health ecosystem. For us to realize AI's maximum impact for all key stakeholders within the healthcare system, we must be fully willing to leverage these new tools by harmonizing data standards, refining algorithms, and creating a more inclusive culture within this space. We suggest the following actionable recommendations.

### Inclusive data collection and utilization

Researchers must advocate for the development and utilization of more comprehensive, diverse, and inclusive datasets that accurately represent marginalized communities and various socioeconomic backgrounds (15, 22, 25–28). There must be collaborations among healthcare institutions, community organizations, and AI developers to ensure the incorporation of diverse perspectives (8, 22, 29, 30).

### Ethical algorithm design and transparency

There is a need to establish stringent ethical guidelines and frameworks for designing AI algorithms. Prioritization of transparency, fairness, and accountability throughout the AI development and implementation process is key (8, 13, 18, 31, 32). We must leverage tools like the AI Fairness Project to mitigate biases and discrimination in AI algorithms used within healthcare.

### Diverse representation in AI development

We must encourage and facilitate greater diversity within AI research and development teams. Inclusive representation from various stakeholders-such as policymakers, healthcare professionals, ethicists, and community representatives- will aid in identifying biases and disparities, ensuring a broader perspective in algorithms development (13, 15, 16, 20, 22, 31, 33–35).

### Establishment of ethical standards

There is a need to formulate and implement clear ethical standards specific to AI applications in healthcare. These standards should focus on reducing biases in AI-driven models, ensuring transparency, and overseeing the responsible development and deployment of AI algorithms to avoid exacerbating disparities (13, 18, 21, 22, 36–39).

### Preservation of human-centric healthcare

There should be an emphasis on the complementary role of AI as a decision support tool rather than a replacement for human interaction in healthcare settings. AI should be designed to enhance, not replace, the clinician-patient relationship, utilizing machine learning and insights to facilitate more informed conversations and personalized care (40).

### Continuous oversight and improvement

Mechanisms must be developed for ongoing evaluation and improvement of AI models. Protocols for regularly auditing and monitoring AI systems must be established to ensure they remain unbiased, transparent, and aligned to reduce health disparities (8, 20, 31, 34, 41–43).

## Discussion: a call to action for shaping an equitable future

Leveraging AI in addressing health disparities necessitates a concerted effort to navigate the challenges while capitalizing on the immense potential these tools offer. By prioritizing inclusive data practices, ethical guidelines, diverse representation, and maintaining a human-centered approach to healthcare, we can harness AI's transformative power to mitigate health disparities and move closer toward achieving health equity for all.

We must implement AI with care, integrity, and an unyielding dedication to equity. Through collaborative action and audacious vision, we can pave the way towards a future where health disparities become less prominent and widespread.
